# A Novel Empowerment System for Patients Living with a Chronic Disease in a Precarious Context

**DOI:** 10.3390/ijerph20010601

**Published:** 2022-12-29

**Authors:** Rita Georges Nohra, Monique Rothan-Tondeur

**Affiliations:** 1 Nursing Sciences Research Chair, Laboratory of Educations and Health Promotion (LEPS), EA 3412, UFR SMBH, F-93017, Sorbonne Paris Nord University, Bobigny, France; 2 Faculty of Public Health, Lebanese University, Fanar, Lebanon; 3 National Institute of Public Health, Clinical Epidemiology and Toxicology-Lebanon, INSPECT-LB, Beirut, Lebanon; 4 Assistance Publique-Hôpitaux de Paris (AP-HP), Nursing Sciences Research Chair, F-75005 Paris, France

**Keywords:** chronic disease, community system, health system, management of chronic disease, patient empowerment, precarious context, therapeutic education, academic system

## Abstract

Nurses play an important role in the management of chronic diseases. Here, we discuss the components of a novel system aimed at empowering patients living with chronic diseases, such as chronic obstructive pulmonary disease (COPD), in a context of precariousness for patients and health personnel, including nurses. This project aimed to evaluate the impact of nursing consultation and remote monitoring on the quality of life in patients with COPD. Two essential elements were linked to promote patient empowerment, which included a network of relationships among the community, hospital, and academic institutions as well as the promotion, contextualization, and co-management of therapeutic education programs among patients. Our results are applicable to all countries with vulnerable populations.

## 1. Introduction

Since the beginning of the 21st century, the increased incidence of noncommunicable diseases (NCDs), also known as chronic diseases, has been responsible for a massive increase in functional disability and mortality worldwide [1]. In 2021, according to the World Health Organization (WHO), NCDs accounted for 74% of all global deaths and 86% of deaths in low- and middle-income countries (LMICs) [1]. Therefore, a change in the management of NCDs is required based on new models of care, such as the chronic care model (CCM) [2].

The CCM is considered the most appropriate and widely used model for managing chronic diseases, particularly diabetes, chronic obstructive pulmonary disease (COPD), and cardiovascular disease, as it recognizes the central role of patients [3]. Since 2015, this model has included the concept of eHealth (eHealth CCM) [4].

The CCM and eHealth CCM were developed as a multidimensional solution to a complex problem. Both models rely on a team of motivated professionals, including nurses, who play a fundamental front-line role in educating patients and ensuring the continuity of care, thereby supporting patient–caregiver relationships and collaborative practices. Despite these advantages, the essential elements of effective collaborations have not yet been identified in these models. The strength-based nursing and healthcare (SBNH) approach, which was developed by Laurie Gottlieb promotes a notion of developing relationships, working with people based on their strengths and abilities, and enhancing individual control and decision-making, thereby highlighting the eight essential values for effective collaboration [5].

However, Beaglehole indicated that most studies on service organizations providing quality chronic care delivery were conducted in high income countries [6]. Therefore, frameworks such as the eHealth CCM may not be directly applicable to LMICs [6] as the challenges faced by these countries are not considered in these models of care.

Herein, we discuss the results of a thesis project entitled “Neith program”, which aimed to improve the quality of life in patients with COPD in Lebanon and evaluate the impact of a nursing consultation and remote monitoring program on their quality of life and skills. First, the literature was reviewed to identify the essential components of a self-management program for patients with COPD. This was the basis for conducting a systematic review-type scoping review. Patient involvement was privileged in the ongoing implementation of this program; therefore, we conducted a phenomenological qualitative study to explore the life experience of these patients in Lebanon. The results of these two studies allowed the development of an intervention plan. To evaluate the feasibility and acceptability of this intervention in the Lebanese population, we conducted a feasibility study.

The results of this program showed that patients with COPD in Lebanon prefer to be actively involved in the management of their disease, but their constant critical living environment, involving health, economics, politics, and security, diminished their capabilities to express their involvement. Capacity alone is inadequate for enabling people to achieve their goals. Positive conversion factors are needed to transform patients’ capacities into capabilities [7]. Based on the results of our research program, a lack of support was identified as a factor hindering the capabilities for disease management of patients with chronic diseases in the Lebanese population. Therefore, we proposed a system that promotes the capabilities of patients with chronic disease living in a precarious context.

This article presents the combination of the eHealth CCM and SBNH along with an overview of the Neith program. Finally, we proposed the components of a system that promote the capabilities of patients living with a chronic disease in a precarious context.

## 2. Discussion

### 2.1. Combination of the eHealth CCM and SBNH

The eHealth CCM and SBNH were selected for this study. Both models were based on the social learning theory (also known as social cognitive theory) of Bandura (1977); the concept of self-efficacy, i.e., the patient has an active role in his/her care, is the central tenet of these models. However, none of the models fully consider this complex process. Although the eHealth CCM requires productive interactions between a team of practitioners and patients, it is not the optimal method to ensure a productive interaction; hence, the requirement for the SBNH approach was indicated by Lorie Gottlieb.

Herein, we discuss McLennan’s (1995) concept of pluralism, which suggests that nursing researchers should consider other theories, concepts, and disciplines to understand a broader perspective of the discipline [8]. Accordingly, the combination of the two models—eHealth CCM and SBNH—provides a more complete description of the chronic disease management process.

In 2011, Kolcaba K. and Kolcaba R. recommended that an integrative method can be used to combine two theories as long as the combination met the eight compatibility criteria. In addition, one or more of the pairings must be based on nursing to ensure a nursing perspective. This caveat is important for ensuring that any research is firmly grounded in the nursing perspective and can be identified as a nursing study rather than simply a study conducted by nurses [9].

Accordingly, the eight heuristic criteria for determining the compatibility of the SBNH with eHealth CCM were examined. These criteria were; shared assumptions, cultural applicability, disciplinary boundaries, nursing education, focus of care, process or product distinction, shared values, and scientific orientation [9].

#### 2.1.1. Shared Assumptions

Both models describe human beings as holistic. The eHealth CCM recognizes the central role of the patient in his or her care, a role that implies responsibility for his or her own health. Similarly, the SBNH views the individual as a unique being who functions as an integrated whole and has the capacity for growth, transformation and self-healing [10].

In both models the person’s environment extends to include family, friends or even community and public resources. The person’s environment according to the SBNH contains powerful energies that act on the particular strengths and deficits that will determine how a person survives or succumbs in a given environment. The eHealth CCM views the person’s environment as the totality of public and community resources that influence the health of individuals [11]. Furthermore, although the eHealth CCM is not a nursing model, it nevertheless emphasizes the importance of productive interactions between the patient and providers. It calls for the involvement of nurses, social workers and patients themselves in the management of their illnesses [3], which is essential to improving health outcomes.

#### 2.1.2. Cultural Applicability

Both theories have universal cultural applicability. They apply to all people regardless of age, beliefs, culture, ethnicity, and socio-demographic level. Neither of them stipulates any restriction.

#### 2.1.3. Disciplinary Boundaries

Although the two models chosen belong to two different disciplines, eHealth CCM from the social discipline and SBNH from the discipline of nursing, they are considered related as they both belong to the humanities and social sciences.

#### 2.1.4. Nursing Education

Laurie Gottlieb’s work focuses on the development of strengths-based nursing as an approach to practice, leadership, management, and education. This approach is the result of an evolution of thinking about what nursing is and how nurses should fulfill their social mandate of health and healing.

The Chronic Care Model does not belong in nursing theory, but patients and health systems around the world have benefited from Ed Wagner’s commitment to transforming healthcare. Best known for its innovation in chronic disease care, the CCM is widely recognized for its ability to guide health care teams in the management of patients with chronic diseases.

#### 2.1.5. Focus of Care

The two models chosen in our study focus closely on chronic disease management. The eHealth CCM and SBNH envision patient learning in a collaborative dynamic that applies to all people regardless of age, culture, ethnicity, and socio-demographic level.

#### 2.1.6. Process or Product Distinction

Both models focus on process outcomes. Indeed, the primary outcome of the eHealth CCM is overall health behavior and effective self-management of chronic disease, while the SBNH envisions a collaborative dynamic in which the nurse understands how a person learns so that learning environments can be developed that allow patients to identify their resources and acquire new ones.

#### 2.1.7. Shared Values

In both models, the ultimate goal is that the patient adopts a self-management posture. Moreover, these models emphasize not only the place of factors purely intrinsic to the person in adopting a behavior, but also other extrinsic factors, notably the environment in which the person evolves and the social relationships that are implemented.

#### 2.1.8. Scientific Orientation

Both models were designed based on Bandura’s social learning theory. As a result, we conclude that the two models are comparable and that the criteria are met for the integration of the eHealth CCM and SBNH.

**Figure 1:**
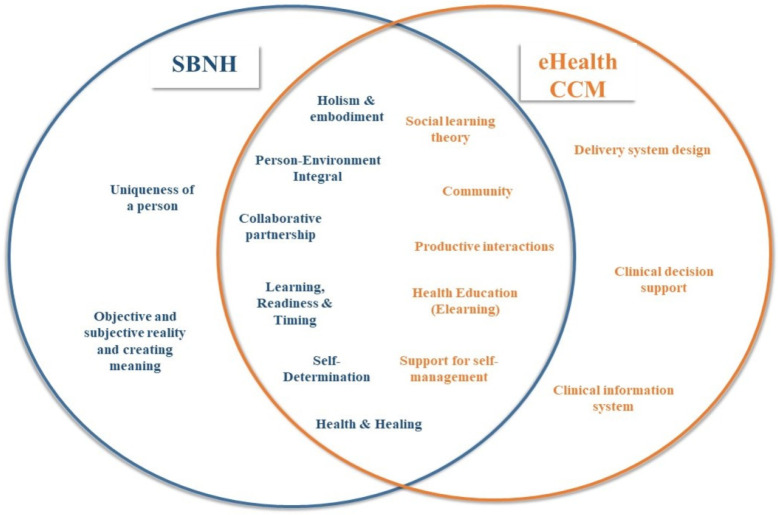
Venn diagram.

A Venn diagram was used to illustrate the association and intersection between these models, thus creating new perspectives (Figure 1).

### 2.2. Results of the Neith Program

The Neith research program aimed to evaluate the impact of nursing consultation and remote monitoring on the quality of life in patients with COPD in Lebanon. Three studies were conducted within the program framework, which led to the development of a patient-centered intervention.

First, a scoping review was conducted to identify the components of a self-management program on patients with COPD. The four components of the self-management programs included in our scoping review were as follows: initiation stage, educational sessions, support, and monitoring methods [12]. Second, a qualitative phenomenological study was conducted to understand the experiences of patients with COPD in Lebanon [13]. The results of these two studies allowed the development of an intervention. A feasibility study was performed to assess the feasibility and acceptability of the intervention in the Lebanese population [14,15].

Throughout the implementation of the Neith program, the eHealth CCM and SBNH were useful for determining objectives, developing research questions and methods, and analyzing results. The purpose of this research program was based on the first concept underlying the SBNH , i.e., “healing and health,” which suggests that illness can be an opportunity to promote health through restoration (healing), learning, and empowerment. In our program, we considered the uniqueness, holism, and indivisibility from a phenomenological perspective, which was the basis for the method of the second study in this program. Similarly, in the intervention, the patient, their family, and nurse developed an education plan that was adapted to the uniqueness of each individual.

However, based on the results of our research program, a lack of support, particularly government support, emerged as a factor limiting the empowerment of patients and thus hindered the capabilities of patients with chronic diseases. Therefore, we proposed various components to strengthen the empowerment of patients with chronic diseases, such as COPD, in a context of precariousness and permanent challenges and crises (economic, security, health, and political).

### 2.3. Components of a System Promoting the Empowerment of Patients with Chronic Disease Living in a Precarious Context

#### 2.3.1. Network of Relationships

**Figure 2:**
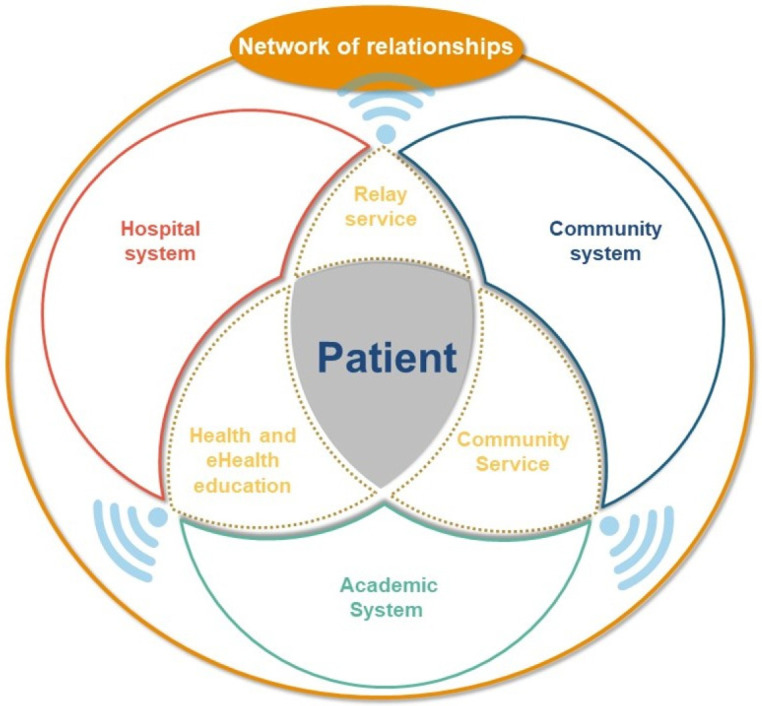
Network of relationships.

Gottlieb stated that a patient who is capable of self-determination is more likely to respect his/her choices and control his/her own health and care decisions. To achieve self-determination, a person must meet the following three requirements: autonomy, willingness to relate, and competence [16]. The fear of discrimination and symptoms of the disease were the most common factors for the withdrawal of patients in our previous qualitative study [13]. The establishment of discussion groups for people with similar problems and construction of a network of relationships are potential solutions. We therefore propose the establishment of a network of relationships and support among the community, hospital, and academic institutions, which revolve around the patient. This network could provide support and encouragement and help the patient integrate into the society. The network can also enhance their ability to receive better medical, behavioral, emotional, and social management of their disease (Figure 2).

##### Relay Services to the Community System

Self-determination refers to the process through which a person makes his/her own decisions [17]; however, the living environment of individuals affects this capacity.

The results of our previous qualitative study showed the extent to which participants living in low-resource settings turn to their families; this is somewhat expected in a cultural context wherein the family is still considered the primary support [13]. This context resulted in the removal of patients from a broader environment due to their limited physical mobility and fear of contamination or being stigmatized by their symptoms [13]. Gottlieb discussed the double-edge of the environment, wherein it can either promote health and support healing or become toxic and detrimental to health [5]. Therefore, community awareness and empowerment are paramount to ensure a supportive environment and friendly community for patients with chronic diseases.

Moreover, the fact that patients mostly relied on their families rather than the community indicates that the awareness of available local governmental and nongovernmental health resources in LMICs such as Lebanon is an important aspect of patient education, as reported by Gagnayre and D’Ivernois [18] and WHO.

The community services in Lebanon have increased owing to the political situation, wherein international aid (budgetary aid or materials) is offered to individuals rather than the government. The challenging economic situation in Lebanon hinders the ability of individuals to obtain medicines. This is because of restricted availability and/or high and nonrefunded prices as well as lack of access to hospital care due to high costs, making it necessary to increasingly link patients to the community through “relay services.” These services can be implemented in primary health care centers or other institutions in the localities. Computerized patient file systems are currently in place in some university hospitals in Lebanon, thereby facilitating the link between these relay services and hospital systems.

**Figure 3:**
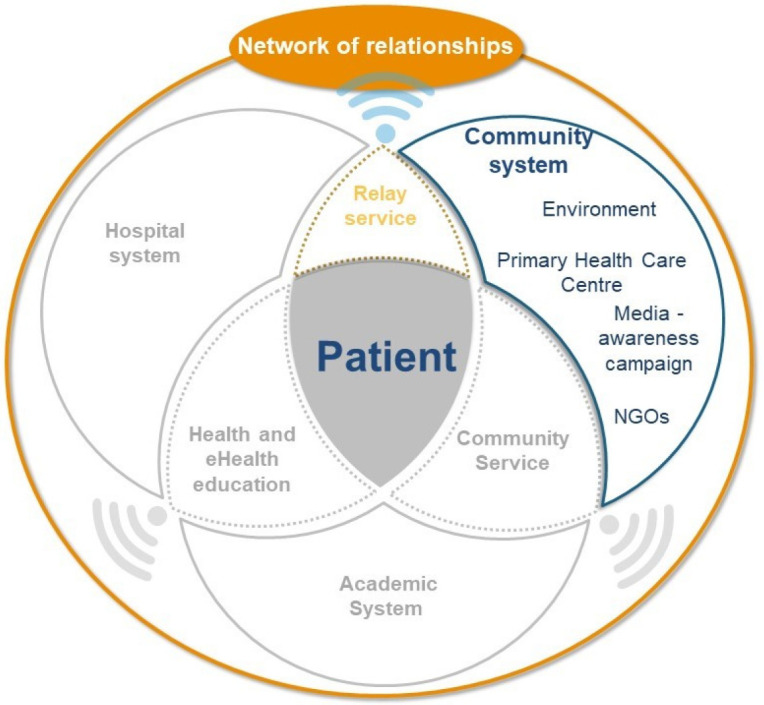
Community system.

Individuals in LMICs such as Lebanon face multiple challenges that can hinder their access to healthcare. In this context, telehealth could help democratize access to healthcare and reduce healthcare costs [19]. Similar to previous research, our feasibility study suggested that monitoring vital signs improves the quality of life and lessens anxiety–depression in patients with COPD, while allowing for prompt patient management [14,20]. Telehealth interventions could link hospitals and “relay services” in the community, promoting easy and rapid sharing of patient health data (Figure 3).

##### Predischarge Education in Hospital Systems

In some precarious contexts, healthcare systems usually emphasize the management of acute illnesses instead of chronic diseases. Moreover, due to budgetary restrictions and staff constraints, the role of nurses in hospitals or primary healthcare centers largely focuses on providing care rather than education for patients; this mostly depends on their workload.

**Figure 4:**
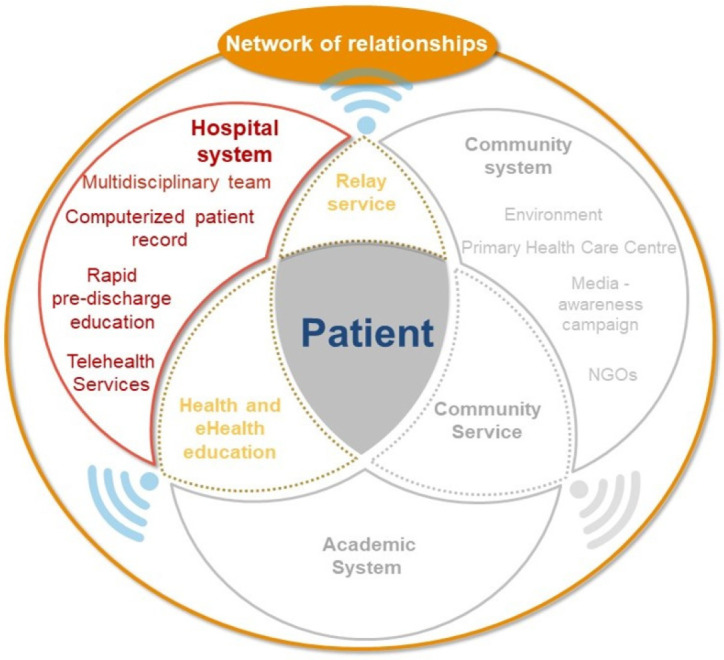
Hospital system.

Currently, a satisfactory care plan cannot be achieved without educational support for the patient and their caregivers. Further, discharging the patient from the hospital without the knowledge and skills necessary for this transition is considered ethically unacceptable [21]. Therefore, it would be essential for hospitals to consider providing therapeutic education as an essential step before patient discharge. Procedures should be established at the hospital level to prepare patients for transfer to their living environment. Moreover, a multidisciplinary team including attending physicians should be established. Local support or potential caregivers should be identified through local relay services at primary healthcare centers (Figure 4).

A recent systematic review highlighted the importance of an approach known as “rapid predischarge education”, which may be effective in facilitating the hospital-to-home transition, thereby increasing patients’ skills and compliance and preventing or reducing early readmissions [21]. According to this approach, exclusive education sessions should be conducted immediately before patient discharge using specific pedagogy provided by trained caregivers [21]. Approximately 50% of the studies included in this review were published in nursing journals, and showed consistent results regarding the educational role of nurses. Thus, the staff should be prepared to provide rapid predischarge education based on their training. Accordingly, it is important for universities to review their curriculum content and approach associated with the training provided to health professionals regarding therapeutic education.

To date, the educational sessions that can provide the best improvement in a therapeutic education program remain unclear. However, the results of our feasibility study revealed that breathing and physical exercises and lifestyle sessions showed the highest acceptability rate [14].

The shortage of nurses has led to an increase in the number of patients assigned to each nurse, which reduces the time devoted to individual patient needs [22] and makes it difficult for nurses to perform interventions. Therefore, it is necessary for hospitals to develop strategies to deal with this problem, such as decreasing the nurse-to-patient ratio or establishing a specialized team to perform such interventions, thereby decreasing the workload of general nurses.

##### Incorporating Community Services within Academic Institutions

A WHO report stated that healthcare professionals must be trained to deliver therapeutic education at two levels: a “basic” level for professionals and an “advanced” level for coordinators delivering therapeutic education [23]. However, a previous study highlighted the lack of preparation among nursing students to engage in educational activities with patients [24]. Based on this observation, it may be necessary to incorporate changes in training programs for nurses and other health professionals, such as medical students, dieticians, and physiotherapists, in terms of the approach and inclusion of telehealth.

**Figure 5:**
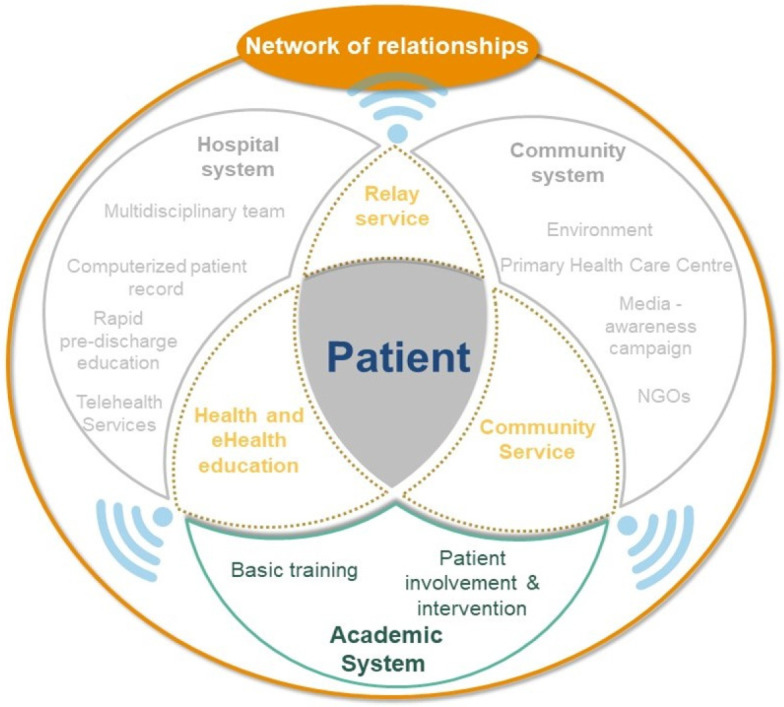
Academic system.

Owing to the impact of universities [25] on the society and environment, it may be essential to develop nursing follow-up services (vital signs, physical assessment, treatment adherence, and healthy lifestyle), including therapeutic education, in university campuses. The development of a setup similar to that already in place for other disciplines in Lebanon, such as dental services and speech therapy, which provide a service to the community while acting as a training ground for students within the university campus, could help provide patient education in nursing schools (Figure 5). Additionally, evaluating patient involvement in these services may be useful as a cofacilitation and peer education method.

#### 2.3.2. Contextualization and Co-Management of Educational Sessions

People with chronic diseases need to reorganize their lives and are confronted with new learning situations related directly to the disease and indirectly to the uncertainty of the disease [26]. Our previous qualitative study revealed that despite living in an environment with limited resources, patients with COPD took responsibility for the management of the disease and their own behaviors, including emotional management and consequences of the disease [13]. In fact, some participants reported self-medication due to financial problems, thereby avoiding medical consultations and hospital admissions. Even in emergency situations, participants used prescriptions that were obtained during previous consultations.

Our findings strongly suggest that in cases of limited resources, learning is primarily based on the experiences of patients living with the disease [13,27]. Accordingly, it is important to prepare patients for managing emergency situations. This requires training of the patient and the co-management of teaching/learning. Therefore, it is necessary to make the patient believe that he/she can be a partner in adopting the scenarios to his/her environment, searching for solutions to problematic situations, and developing his/her commitment.

## 3. Conclusions

Based on the results of the research program Neith, a lack of government support emerged as a factor limiting the empowerment of patients with chronic diseases. Therefore, we proposed establishing a network of relationships that ensures collaborations with the patient as a basic component of a system promoting the empowerment of patients with chronic diseases, in a context of precariousness and permanent challenges and crises (economic, security, health, and political). We suggest implementing relay services to the community using telehealth to facilitate the link between the hospital and the community and incorporating community services within academic institutions, which could provide a service to the community while acting as a training ground for students within the university campus.

## 4. Limitations and Further Research

The components proposed in this article are the result of the authors’ reflections on the axes that could strengthen the empowerment of patients with chronic diseases, in a context of precariousness and permanent challenges. Other elements could be added to give this proposal a more complete perspective.

Encouraging governments and policy makers to implement new models for linking hospitals, communities, and academic institutions to achieve better quality of life in patients with chronic diseases remains a challenge. To fulfill the expectations of patients living with chronic diseases such as COPD, it is necessary to increase the awareness of the need for systematic approaches among political authorities and health professionals.

Future research is warranted to evaluate the impact of models such as the eHealth CCM on the quality of life in patients living with a chronic disease in a precarious context and to assess the practical implementation of new programs to overcome these challenges.
